# Expression of Immunomodulatory Checkpoint Molecules in Drug-Resistant Neuroblastoma: An Exploratory Study

**DOI:** 10.3390/cancers14030751

**Published:** 2022-01-31

**Authors:** Nicholas J. Skertich, Fei Chu, Imad A. M. Tarhoni, Stephen Szajek, Jeffrey A. Borgia, Mary Beth Madonna

**Affiliations:** 1 Department of Surgery, Division of Pediatric Surgery, Rush University Medical Center, Chicago, IL 60612, USA; Fei_Chu@rush.edu (F.C.); sszajek19@gmail.com (S.S.); 2 Department of Pathology, Rush University Medical Center, Chicago, IL 60612, USA; imad_tarhoni@rush.edu (I.A.M.T.); jeffrey_a_borgia@rush.edu (J.A.B.); 3 Department of Cell & Molecular Medicine, Rush University Medical Center, Chicago, IL 60612, USA

**Keywords:** neuroblastoma, doxorubicin, drug resistance, programmed death ligand 1, checkpoint protein molecules, checkpoint molecule inhibition

## Abstract

**Simple Summary:**

Neuroblastoma is a common childhood cancer with poor prognosis. Prior studies suggest that inhibition of molecules called checkpoint proteins, which normally prevent one’s own immune system from attacking itself, has been successfully used for treatment of multiple advanced adult cancers but has yet to be fully explored in neuroblastoma. Cancer can hijack these pathways to prevent the immune system from recognizing and destroying cancer cells. We investigated checkpoint protein expression in pediatric neuroblastoma and its role in drug resistance. We created drug-resistant neuroblastoma cell lines and compared expression of checkpoint proteins between drug-resistant and parental cell lines. In total, 13 checkpoint proteins were expressed by all cell lines regardless of drug resistance. Although PD-L1 and checkpoint proteins do not necessarily impart drug resistance, they may be potential targets for drug therapy. Benchmarking checkpoint proteins provides the basis for future studies identifying targets for directed therapy and biomarkers for cancer detection or prognosis.

**Abstract:**

Neuroblastoma is a common childhood cancer with poor prognosis when at its advanced stage. Checkpoint molecule inhibition is successful in treating multiple advanced adult cancers. We investigated PD-L1 and other checkpoint molecule expression to determine their roles in drug resistance and usefulness as targets for drug therapy. We developed three doxorubicin-resistant (DoxR) cell lines from parental cell lines. Matrigel in vitro invasion assays were used to compare invasiveness. Western blot assays were used to compare PD-L1 expression. Immuno-oncology checkpoint protein panels were used to compare concentrations of 17 checkpoint molecules both cellular and soluble. PD-L1 and 12 other checkpoint molecules were present in all cell lysates of each cell line without significantly different levels. Three were solubilized in the media of each cell line. PD-L1 is expressed in all DoxR and parental neuroblastoma cells and may be a potential target for drug therapy although its role in drug resistance remains unclear. Benchmarking checkpoint molecules provides the basis for future studies identifying targets for directed therapy and biomarkers for cancer detection or prognosis.

## 1. Introduction

Neuroblastoma is a common solid malignancy in childhood that often presents with late-stage disease. Therefore, it accounts for up to 15% of childhood cancer deaths with less than 40% survival despite aggressive therapy [1,2]. For intermediate- and high-risk neuroblastoma, patients often require chemotherapy with doxorubicin as a key component [1,2]. Unfortunately, resistance and relapse are common, making a cure difficult to achieve. Despite therapeutic advances, new treatments are still urgently needed for advanced disease.

A novel method successful in the treatment of chemotherapy-resistant cancers in adults is inhibiting immunomodulatory checkpoint molecules (ICMs), which normally serve to prevent the immune system from reacting against healthy cells [3,4,5]. However, these regulatory pathways can be hijacked by cancer too, which prevents one’s immune system from recognizing and destroying it [5]. The programmed death 1 (PD-1)–programmed death ligand 1 (PD-L1) interaction is one of the best-studied pathways. When PD-L1 expressed on cancer cells interacts with PD-1, a tyrosine-kinase receptor protein expressed by B and T lymphocytes, lymphocyte proliferation is inhibited, leading to cancer survival [6,7,8]. Inhibitors of PD-1, PD-L1 and cytotoxic T-lymphocyte-associated protein 4 (CTLA-4) are currently and successfully used in the treatment of non-small-cell lung cancer, advanced melanoma, and Hodgkin’s lymphoma, among others [9,10,11,12,13]. Immunotherapies that inhibit other checkpoint pathways are also under investigation including drugs that inhibit lymphocyte-activation gene-3 (LAG-3), B- and T-lymphocyte attenuator (BTLA) and T-cell immunoglobulin-3 (TIM-3) [5,14,15,16,17,18].

In regard to pediatric solid tumors, inhibition of checkpoint molecules as targeted drug therapy is still in the early stages. Although a few phase I/II trials are underway, preliminary studies have demonstrated conflicting evidence in regard to which checkpoint molecules are expressed across the various tumor types [16,19,20,21,22,23,24]. Regarding neuroblastoma, a few trends have emerged and mouse models for targeted inhibition of the PD-1–PD-L1 pathway have been developed. First, PD-L1 is expressed in varying levels among patients with NB and seems to portend a worse prognosis [24,25,26,27]. Second, in murine models, PD-L1 inhibition does indeed lead to tumor cell death but is model dependent and may not have a prolonged effect [28,29,30,31,32]. Third, in combination with CTLA-4 or other chemotherapeutic regimens, a prolonged response may be seen [32,33]. These data suggest that blockage of ICMs may provide viable treatment regimens in pediatric neuroblastoma. However, prior research has fallen short in terms of scope of checkpoint molecules explored and evaluation across patients with chemotherapy-resistant or advanced disease.

Our goal was to explore PD-L1 expression of cell lines of doxorubicin resistance (DoxR) neuroblastoma to investigate its role as a mechanism for drug resistance. Our prior research with doxorubicin-resistant osteosarcoma suggests a correlation between checkpoint molecule expression and drug resistance [34]. This has also been demonstrated in a few adult cancers such as squamous cell carcinoma of the neck and prostate cancer [35,36]. We also aimed to identify and benchmark additional checkpoint proteins to direct future studies on drug resistance and metastasis mechanisms, for use as potential biomarkers for diagnosis and prognosis, and for possible drug targets. We hypothesized that drug-resistant cell lines would have greater expression of ICMs.

## 2. Materials and Methods

### 2.1. Cell Lines

The SKN-SH, SKN-AS, and SKN-DZ cell lines were purchased from the American Type Culture Collection (Manassas, VA, USA). SKN-SH is a human neuroblastoma cell line derived from brain tissue of a 4-year-old female. SKN-AS is human neuroblastoma derived from brain tissue of a 6-year-old female. SKN-DZ is human neuroblastoma derived from brain tissue of a 2-year-old female [37].

### 2.2. Reagents

Dulbecco’s modified Eagle’s medium (DMEM) and heat-inactivated fetal bovine serum (FBS) were obtained from Fisher Scientific (Chicago, IL, USA). Penicillin and streptomycin were obtained from HyClone (Logan, UT, USA). Doxorubicin was obtained from Sigma (St. Louis, MO, USA). 3-(4,5-dimethyl-2-thiazolyl)-2,5-diphenyltetrazolium bromide (MTT) was purchased from Thermo Fisher Scientific (Asheville, NC, USA). Rabbit monoclonal antibodies for PD-L1 (clone E1L3N^®^) were obtained from Cell Signaling Technology (Danvers, MA, USA), and monoclonal mouse anti-β-actin (clone AC-15) from Sigma (St. Louis, MO, USA). Secondary goat anti-rabbit immunoglobin G (IgG)-HRP (W401B) and goat anti-mouse IgG-HRP (W402B) monoclonal antibodies were purchased from Promega (Madison, WI, USA). Enhanced chemiluminescence reagents were obtained from Thermo Fisher Scientific (Asheville, NC, USA).

### 2.3. Cell Culture, Drug Treatment and Cytotoxicity Assay

All cell lines were maintained in complete media, which consisted of DMEM with 10% heat-inactivated FBS, 100 units/mL penicillin and 100 µg/mL streptomycin and grown in a humidified chamber (37 °C, 5% CO_2_). Doxorubicin-resistant (DoxR) cells were generated by incubating parental WT cells with incremental concentrations of doxorubicin ranging from 1 nM to 1 µM over a six-month period. Treatment began with 1 nM and was increased to the next 10-fold increment after surviving five consecutive passages. Cells were considered to be resistant after surviving five consecutive passages in 1 µM doxorubicin. Cell viability was determined by the quantitative colorimetric MTT assay according to Roche (previously Boehringer Mannheim) and purchased from Sigma Aldrich (St. Louis, MO, USA) as previously described [38]. For cell culture, drug treatment, and MTT assay, three separate experiments were run. Within each experiment, samples were run in triplicate.

### 2.4. In Vitro Invasion Assay

Cell invasion was determined and analyzed using a membrane invasion culture system purchased from Fisher Scientific (Chicago, IL, USA). The number of cells able to invade through a membrane coated with the defined Matrigel extracellular matrix during a 24 h period was compared to the number counted using a control insert with no Matrigel. Cells were seeded at 2.5 × 10^4^ and incubated for 24 h. Cells that migrated through the membrane were fixed and stained with a Diff-Quik staining kit obtained from Electron Microscopy Sciences (Hatfield, PA, USA). Three fields at 40× magnification were counted by light microscopy (technical replicates) for each experiment. Three biologic experiments were conducted (therefore nine replicates in total). Invasion was reported as the number of cells on the membrane divided by the number on the control membrane (mean ± standard error).

### 2.5. SDS-PAGE and Western Blot

Parental and DoxR cells were seeded in complete medium and cultured for 48 h. Cells were lysed using NP40 Cell Lysis Buffer purchased from Thermo Fisher Scientific (Asheville, NC, USA) with Protease Inhibitor Cocktail obtained from Sigma-Aldrich (St. Louis, MO, USA). Total protein concentration was determined using the bicinchoninic acid assay (BCA) assay from Thermo Fisher Scientific (Asheville, NC, USA) using the supplied albumin as the analytical standard. Equal amounts of protein were reduced in 1× sample buffer (Laemmli) from Bio-Rad (Hercules, CA, USA), with 5% β-mercaptoethanol from Fisher Scientific (Chicago, IL, USA) boiled for five minutes, separated by electrophoresis on 4–20% Mini-Protean TGX Precast Protein Gels obtained from Bio-Rad (Hercules, CA, USA) and transferred using the Invitrogen iBlot 2 Gel Transfer Device purchased from Thermo Fisher Scientific (Asheville, NC, USA), onto nitrocellulose membranes via iBlot 2 Transfer Stacks also purchased from Thermo Fisher Scientific (Asheville, NC, USA). Proteins of interest were identified with specific primary antibodies followed by HRP-conjugated secondary antibodies. Immunoreactive bands were detected by chemiluminescence with image capture on an iBright CL 1500 Imaging System bought from Thermo Fischer Scientific (Asheville, NC, USA). Three separate Western blot experiments were conducted.

### 2.6. Human Immuno-Oncology Checkpoint Protein Panel

Proteins from cell lysates, lysed using RIPA buffer from Thermo Fisher Scientific (Asheville, NC, USA) in a 10% protease inhibitor cocktail (as above), were tested for 17 checkpoint proteins using the Human Immuno-Oncology Checkpoint Protein Panel purchased from MilliporeSigma (St. Louis, MO, USA). These were chosen based on prior work with doxorubicin-resistant osteosarcoma and the commercial availability of the panel [34]. Similarly, the media from cell culture removed directly from the cell culture dishes after cells were plated for 24 h, for each cell line was collected and tested for the same 17 checkpoint proteins in a similar fashion. The 17 checkpoint protein molecules evaluated included PD-1, PD-L1, programmed death ligand 2 (PD-L2), CTLA-4, LAG-3, TIM-3, BTLA, cluster of differentiation 27 (CD27), cluster of differentiation (CD28), cluster of differentiation 40 (CD40), cluster of differentiation 80 (CD80), cluster of differentiation 87 (CD86), herpesvirus entry mediator (HVEM), inducible T-cell costimulatory (ICOS), glucocorticoid-induced TNFR-related protein (GITR), ligand for receptor TNFRSF18/AITR/GITR (GITRL), and Toll-like receptor 2 (TLR-2). All primary data points were collected via the Luminex FLEXMAP 3D system from Lumine Corporation (Austin, TX, USA), and protein concentrations were calculated using a five-parametric fit algorithm xPONENT v4.0.3 by Luminex Corp (Austin, TX, USA). Three separate experiments were conducted and all samples were run in triplicate using lysates or media from different passages.

### 2.7. Statistical Analysis

For in vitro invasion assays, categorical variables were compared between groups using chi-square tests. For Western blot analysis, differences between parental and DoxR cell lines were assessed using Student’s unpaired *t*-tests. For PD-L1 expression in tumor samples across stages, a Kruskal–Wallis test was used. To compare expression of checkpoint molecules between parental and DoxR cell lines (using results from the Human Immuno-Oncology Checkpoint Protein Panel), Wilcoxon rank-sum tests were used. Statistical differences were determined using *p* < 0.05 via SPSS 26 (Armonk, NY, USA).

## 3. Results

### 3.1. Doxorubicin-Resistant Cells Are More Invasive Than Their Parental Cells

**Figure 1:**
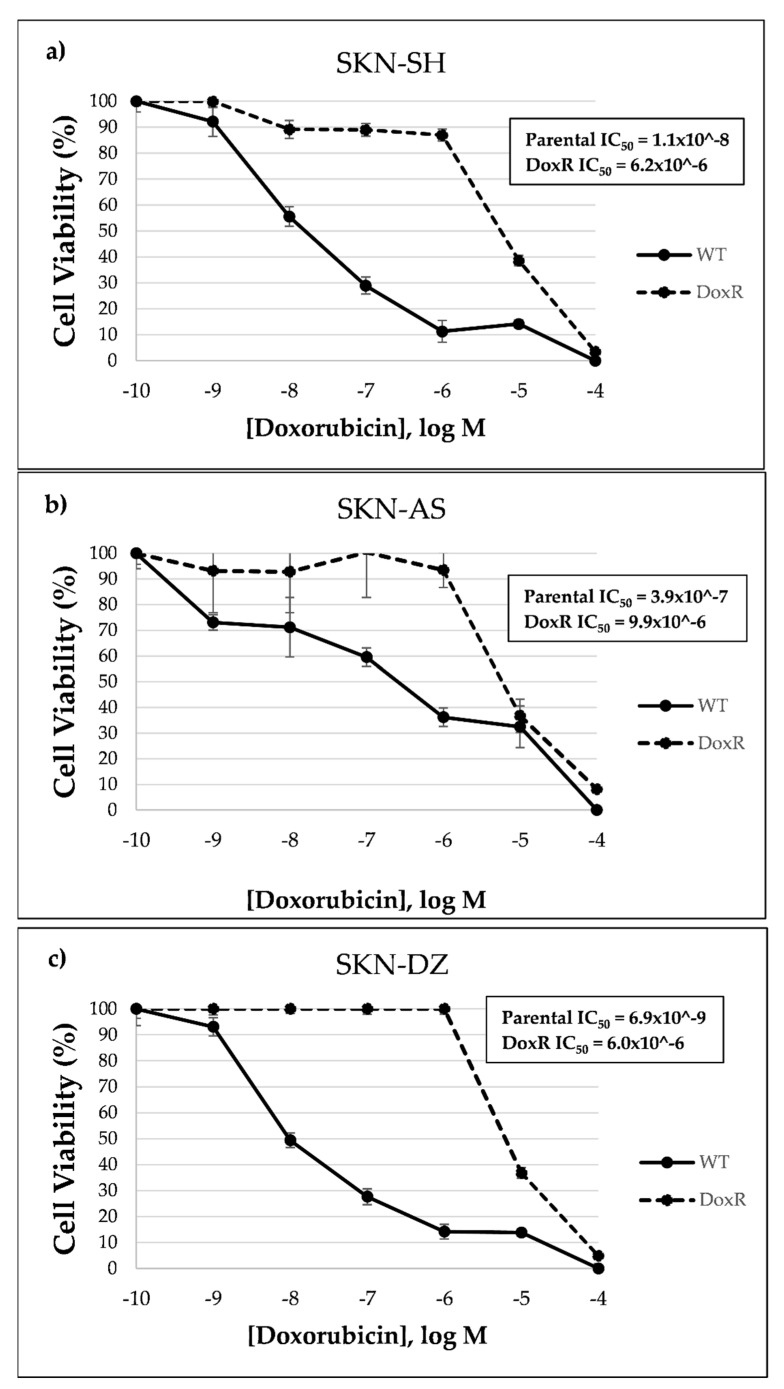
Doxorubicin-resistant cell lines are resistant compared to parental cell lines based on cell viability assays: (**a**) SKN-SH, (**b**) SKN-AS, and (**c**) SKN-DZ.

Doxorubicin-resistant cell lines were more resistant than their parental cells as determined by a greater than 100-fold difference in half maximal inhibitory concentration (IC50) than their parental, doxorubicin-sensitive, cell lines based on MTT assays, as shown in Figure 1.

**Figure 2:**
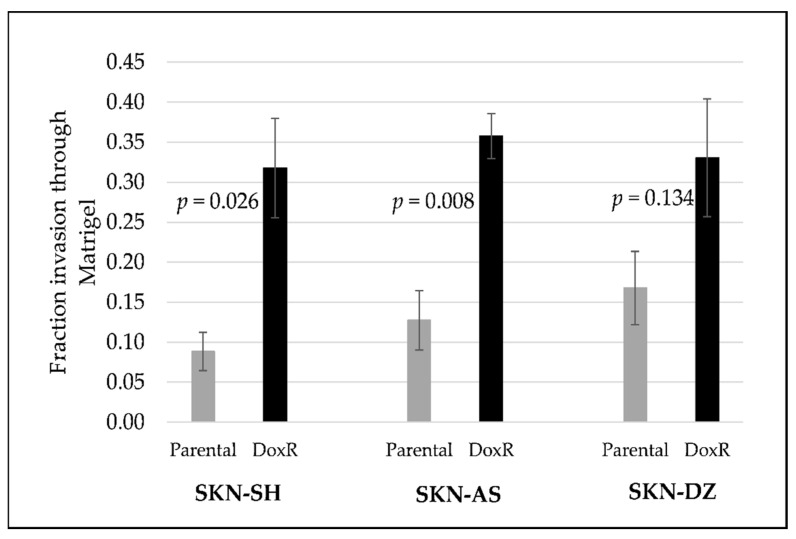
Matrigel in vitro invasion assay (mean and standard error) demonstrating doxorubicin-resistant (DoxR) cell lines are more invasive compared to their parental cell lines: SKN-SH, SKN-AS and SKN-DZ.

The invasiveness of the SKN-SH, SKN-AS, and SKN-DZ parental and DoxR cell lines was determined using Matrigel invasion assays and compared. For each cell line, the DoxR cells were more invasive than their parental cell lines. SKN-SH DoxR cells were significantly more invasive than their parental cells (fraction of invasion 0.32 vs. 0.09, *p* < 0.026) as were SKN-AS DoxR cells compared to parental (0.36 vs. 0.13, *p* = 0.008). SKN-DZ DoxR cells were not significantly more invasive than parental cells (0.33 vs. 0.17, *p* = 0.134), as shown in Figure 2.

### 3.2. Neuroblastoma Expresses PD-L1 Regardless of Doxorubicin Resistance

**Figure 3:**
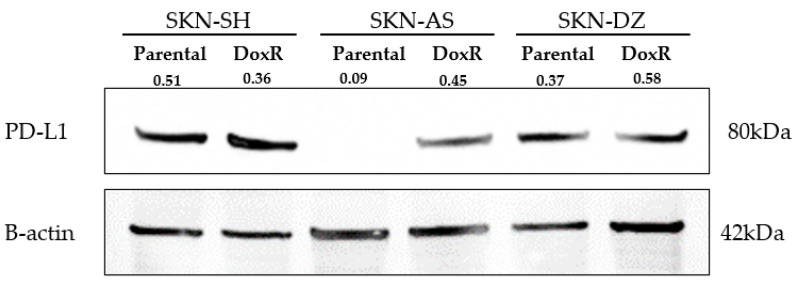
Western blot demonstrating PD-L1 expression across SKN-SH, SKN-AS, and SKN-DZ parental and doxorubicin-resistant (DoxR) cell lines. Numbers indicate relative band intensities of PD-L1 protein normalized to β-actin. The uncropped western blot figures were presented in Figure S1.

The PD-L1 protein level from whole-cell lysates was similar between SKN-SH DoxR and parental cell lines as well as SKN-DZ DoxR and parental cell lines but was higher in the SKN-AS DoxR cell line than its parental cell line, as shown in Figure 3.

### 3.3. Neuroblastoma Expresses Multiple Additional Checkpoint Molecules Both Cellular and Soluble

**Figure 4:**
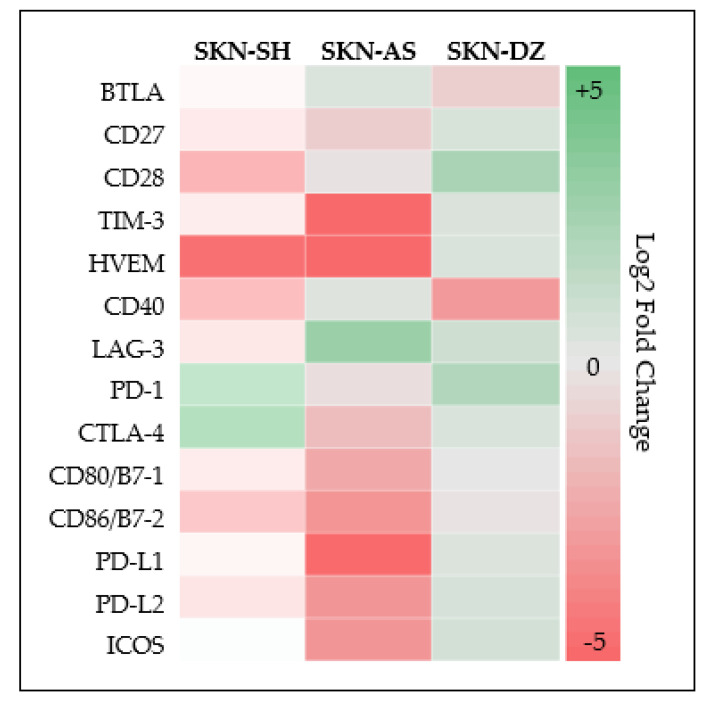
Heat map of log2-fold changes in concentration of each checkpoint protein between doxorubicin-resistant compared to parental cell lines.

Cell lysates of SKN-SH, SKN-AS, and SKN-DZ DoxR and parental cell lines each expressed 13 out of the 17 checkpoint molecules for which we tested without significant difference between DoxR and parental cells for any checkpoint protein across all cell lines, as shown in Table 1. Checkpoint proteins expressed included BTLA, CD27, CD28, TIM-3, HVEM, CD40, GITR, LAG-3, CD80, CD86, PD-L1, PD-L2, and ICOS. In addition, SKN-SH and SKN-AS parental cell lines also expressed PD-1 and CTLA-4. Log2-fold changes between DoxR and parental cells for each protein expressed across each cell line are summarized via heat map in Figure 4. Conditioned media from culture of both DoxR and parental cells across each cell line contained CD40, LAG-3, and PD-L2 without significant differences between DoxR and parental cells, as shown in Table 2. SKN-SH DoxR cells also secreted CD80, CD86 and PD-L1 into the cell media.

## 4. Discussion

This study is the first to explore cellular and soluble ICM protein expression in chemotherapy-resistant neuroblastoma cell lines. We successfully created drug-resistant neuroblastoma cell lines that were more invasive than their parental cell lines serving as a proxy for advanced disease. Next, we evaluated and confirmed PD-L1 expression in each drug-resistant and parental cell line, demonstrating it unlikely plays a role in facilitating drug resistance. Finally, 12 more ICMs were found to be expressed by all neuroblastoma cell lines, three of which were also secreted into the cell culture media. Benchmarking these ICMs may lay the foundation for other studies to explore checkpoint molecules as biomarkers for disease detection or prognosis or for directed drug therapy.

The expression of PD-L1 in each parental and DoxR neuroblastoma cell line suggests that the PD-1–PD-L1 pathway may not play a direct role in the development of drug resistance. Rather, these cell lines likely all express PD-L1, as a mechanism of immune escape and possibly indicate advanced disease rather than a mechanism for drug resistance. However, the fact that it is expressed across all cell lines may make it a promising target for directed drug therapy. This particular protein was chosen because there are already commercially available drugs on the market that target the interaction between PD-1 and PD-L1 for adult cancers. Moreover, preliminary studies regarding its expression and inhibition in neuroblastoma have had conflicting results whether PD-L1 is expressed and if its inhibition leads to tumor regression. Aoki et al. initially reported no PD-L1 expression in NB patients, but was contradicted by Chowdhury et al. who reported a high level of PD-L1 expression which positively correlated with a worse survival [23,24]. Since that time, other studies have confirmed its expression consistently in vitro [25,31,33,39,40,41]. Unfortunately, evaluation of patient tumor tissue has had limited success, but expression does seem to correlate with prognosis [24,25,26,27,40]. Therefore, we hypothesized that PD-L1 expression may play a role in drug resistance. In this study, we did not consistently identify differing levels of PD-L1 expression between parental and DoxR cell lines and its role as a mediator of drug resistance seems less likely. Conversely, since PD-L1 was expressed in both parental and DoxR cell lines, it still may be promising as a marker of advanced disease or target for directed drug therapy.

The successful treatment of cancer with inhibition of the CTLA-4, PD-1, PD-L1, and CD80/86 pathways in adults was the basis for our exploration into additional checkpoint molecule expression in neuroblastoma. By better understanding the expression of these proteins in the tumor microenvironment and how they control immune suppression may expand their usefulness as biomarkers or targets for drug therapy into the pediatric population. There are multiple advantages to using our drug-resistant and parental cell lines. It preserves our biorepository, it can direct future study of checkpoint pathways, and comparison between parental and drug-resistant groups may identify mechanisms of drug resistance and metastasis.

In total, 15 of 17 ICMs were measurable in this study, 13 in all parental and DoxR cell lines. Similar to our results with PD-L1 via Western blot analysis, there were no significantly different levels between the groups when using our checkpoint protein panel as visually demonstrated in the heat map, as shown in Figure 4 Therefore, while conclusions as to their role in mediating drug resistance are difficult to make, measurable expression across all cell lines still provides valuable information. All cell lines expressed PD-L1 and there are currently six FDA-approved drugs inhibiting the PD-1–PD-L1 pathway on the market [42]. Moreover, all cell lines expressed CD80/86 and two of the parental cell lines expressed CTLA-4. Currently there is one FDA-approved drug inhibiting the CTLA-4, CD80/86 pathway, where CD86 in particular, when bound to CTLA-4, serves as a costimulatory molecule inhibiting naïve and memory T-cell activation [42,43]. Multiple other adult clinical trials are underway using novel checkpoint inhibitors including 10 evaluating the use of anti-LAG-3 antibodies, and three studying anti-TIM-3 [44]. A recent in vivo study of ovarian carcinoma in mice concluded and demonstrated a survival benefit to using BTLA inhibition [45]. LAG-3, TIM-3, and BTLA are expressed across all cell lines. Our study expands the knowledge of checkpoint molecule activity in the tumor microenvironment and provides translatable data to direct further in vitro and in vivo studies of ICMs not previously known to be expressed by neuroblastoma, especially as novel immunomodulatory therapies become FDA approved.

Lastly, we measured the checkpoint protein levels in the cell culture media. Molecules can be shed from cancer cells as a result of exosomal or proteolytic cleavage of membrane-bound forms. In regard to checkpoint molecules, this can induce immunosuppression and cancer survival [46,47]. In osteosarcoma, another common pediatric solid tumor, PD-L1 seems to be mediated by exosomal shedding [48]. In other cancers such as esophageal adenocarcinoma or invasive ductal carcinoma breast cancer, PD-L1 is shed via proteolytic cleavage [47,49]. Regardless, levels of these soluble markers may vary based on the health of the patient and advancement of the disease with multiple studies demonstrating the utility of circulating levels of PD-L1 for prognosis including a recent meta-analysis of adult solid tumors [50,51]. In neuroblastoma, there are no studies we are aware of evaluating the shedding of immune checkpoint molecules. It is known that gangliosides are overexpressed and actively shed, but their use as biomarkers although promising, has yet to come to fruition [52,53]. However, identification of soluble markers has the potential to be useful for early disease detection, identification of disease relapse, assisting with prognosis, or directing future studies. Here, within, we identified CD40, LAG-3 and PD-L2 solubilized in the cell culture media across all cell lines. Once again, we did not find significantly different levels between the parental cell lines and DoxR cell lines, although the trend seems to demonstrate higher levels in DoxR cell lines. We also only detected soluble PD-L1 in SKN-SH DoxR cells. These results are still promising since we confirmed neuroblastoma has soluble ICMs. However, additional studies are needed to better evaluate their utility and to draw more concrete conclusions.

This study was not without limitations. First, although there appeared to be different expression of PD-L1 in the parental SKN-AS and DoxR SKN-AS cell lines on Western blot, both expressed PD-L1 when measured via checkpoint protein panel, which is more sensitive and specific. The discrepancy may be secondary to the higher-than-expected molecular weight of PD-L1 on Western blot; however, this molecular weight is not inconsistent with other studies using the same antibody [54]. In addition, we were limited in the depth of our checkpoint exploration. We used three cell lines, but we plan to expand testing to patient tissue and serum samples which may better elucidate the use of ICMs for prognosis and directed therapy. Finally, as an exploratory study, we did not evaluate inhibition of PD-L1 or other checkpoint molecules in vivo since the goal was to lay the groundwork for future research. However, this would be useful to determine the clinical utility of inhibiting these ICMs.

## 5. Conclusions

This is one of the first studies to evaluate checkpoint molecule expression in drug-resistant neuroblastoma. We demonstrated that PD-L1 is expressed across all cell lines without significant change between parenteral and drug-resistant cell lines; therefore, expression may not be related to drug resistance. We also demonstrated that many other checkpoint molecules are expressed by neuroblastoma in the tumor microenvironment and that some of these are secreted into the cell culture media. Therefore, they may still be useful as targets for further directed drug therapy research or as cancer biomarkers.

## Figures and Tables

**Table 1:** Summary of immune checkpoint proteins present in parental versus doxorubicin-resistant (DoxR) neuroblastoma cell lysates from SKN-SH, SKN-AS, and SKN-DZ cell lines. Values are reported as medians in pg/mL/mg of cellular protein.

Cell Line	SKN-SH			SKN-AS			SKN-DZ		
Target	Parenteral	DoxR	*p*-Value	Parenteral	DoxR	*p*-Value	Parenteral	DoxR	*p*-Value
BTLA	36.1	31.3	1	1136.0	32.1	0.2	99.3	52.9	0.667
CD27	4.9	3.0	0.4	1.9	2.6	0.4	2.8	4.3	0.4
CD28	11.4	2.1	0.1	6.2	3.1	0.8	3.3	17.4	0.5
TIM-3	3.5	2.3	0.1	2.5	2.2	0.7	2.2	3.2	0.4
HVEM	0.3	0.0	0.1	97.8	0.1	0.5	0.1	0.2	0.1
CD40	15.8	3.6	0.7	1820.0	23.1	0.1	6.5	0.8	0.4
GITR	4.4	4.0	0.1	2.4	3.1	0.1	2.7	4.0	0.1
LAG-3	107.1	62.0	0.1	34.0	63.7	0.4	45.8	90.5	0.7
TLR-2	<LLoQ	<LLoQ		<LLoQ	<LLoQ		<LLoQ	<LLoQ	
GITRL	<LLoQ	<LLoQ		<LLoQ	<LLoQ		<LLoQ	<LLoQ	
PD-1	0.8	<LLoQ		2.8	<LLoQ	0.1	<LLoQ	<LLoQ	
CTLA-4	0.4	<LLoQ		2.0	<LLoQ	0.7	<LLoQ	<LLoQ	
CD80/B7-1	2.2	1.4	0.4	3.5	1.1	0.1	1.4	1.5	0.7
CD86/B7-2	2.9	0.8	0.1	4.4	0.8	0.1	1.1	1.0	1
PD-L1	4.5	3.6	0.7	29.5	3.1	0.1	2.8	3.8	0.7
PD-L2	8.4	4.7	0.1	246.8	8.0	0.1	5.8	9.0	0.2
ICOS	15.0	16.4	0.7	153.9	16.4	0.1	10.9	19.1	0.2

**Table 2:** Summary of immune checkpoint proteins present in parental versus doxorubicin-resistant (DoxR) neuroblastoma cell media from SKN-SH, SKN-AS, and SKN-DZ cell lines. Media was obtained after cells were plated for 24 h. Values are reported as medians in pg/mL/mg of cellular protein.

Cell Line	SKN-SH			SKN-AS			SKN-DZ		
Target	Parenteral	DoxR	*p*-Value	Parenteral	DoxR	*p*-Value	Parenteral	DoxR	*p*-Value
CD40	3.205	4.65	0.667	16.97	1.34	0.121	0.285	1.83	0.121
LAG-3	24.755	32.62	1	17.13	32.28	0.439	11.12	71.33	0.121
CD80/B7-1	<LLoQ	0.75	N/A	<LLoQ	<LLoQ	N/A	<LLoQ	<LLoQ	N/A
CD80/B7-2	<LLoQ	0.04	N/A	<LLoQ	<LLoQ	N/A	<LLoQ	<LLoQ	N/A
PD-L1	<LLoQ	0.57	N/A	<LLoQ	<LLoQ	N/A	<LLoQ	<LLoQ	N/A
PD-L2	3.19	2.42	0.121	40.38	2.095	0.121	1.06	3.65	0.121

## Data Availability

The data presented in this study are available on request from the corresponding author.

## Supplementary Materials

The following are available online at https://www.mdpi.com/article/10.3390/cancers14030751/s1, Figure S1: the uncropped western blot figures.
