# Supplementary material for: Expression of Immunomodulatory Checkpoint Molecules in Drug-Resistant Neuroblastoma: An Exploratory Study
Source: Cancers (Basel). 2022 Jan 31;14(3):751. doi: 10.3390/cancers14030751 (PMC8833944; doi:10.3390/cancers14030751)
Supplement: Supplementary file 1 [file cancers-14-00751-s001.zip › cancers-1558154-supplementary.pdf]

Supplementary Materials: Expression of Immunomodulatory Checkpoint Molecules in Drug-Resistant Neuroblastoma: An Exploratory Study

Nicholas J. Skertich, Fei Chu, Imad A.M. Tarhoni, Stephen Szajek, Jeffrey A. Borgia and Mary Beth Madonna

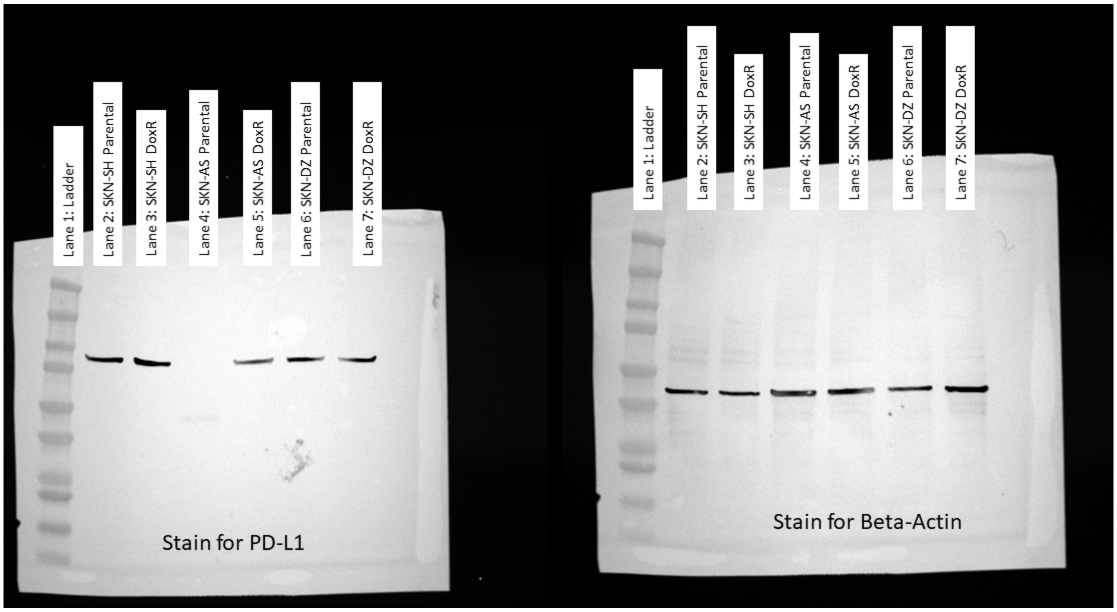

Figure S1. the uncropped western blot figures.
